# Responses of a common tropical epiphyte, *Asplenium nidus*, to changes in water and nutrient availability

**DOI:** 10.1093/aobpla/plad076

**Published:** 2023-11-09

**Authors:** Xiao-Zhen Chen, J Aaron Hogan, Chiao-Ping Wang, Pei-Ling Wang, Teng-Chiu Lin

**Affiliations:** Department of Life Science, National Taiwan Normal University, Taipei 11677, Taiwan; Department of Biology, University of Florida, Gainesville, FL 32611, USA; Silviculture Division, Taiwan Forestry Research Institute, Taipei 10066, Taiwan; Institute of Oceanography, National Taiwan University, Taipei 10617, Taiwan; Department of Life Science, National Taiwan Normal University, Taipei 11677, Taiwan

**Keywords:** *Asplenium nidus*, atmospheric deposition, climate change, co-limitation, epiphytes, nutrient limitation, water stress

## Abstract

Epiphytes are highly dependent on atmospheric inputs of water and nutrients. Reductions in water availability associated with warming and climate change and continual atmospheric nitrogen (N) deposition can affect plant growth but few studies have evaluated the effects of changes in both water and nutrient availabilities on epiphytes. We experimentally tested whether epiphyte growth is more water- or nutrient-limited, if nutrient limitation was stronger for nitrogen or phosphorus, and whether nutrient limitation interacts with water availability. We applied watering (high and low) and nutrient addition (control, +N, +P, +N+P) treatments to greenhouse-grown *Asplenium nidus*, a common epiphytic fern found in many tropical and subtropical wet forests. We measured leaf area production and leaf elemental concentrations to assess how *A. nidus* growth and physiology respond to changes in water and nutrient availabilities. We found that leaf growth of *A. nidus* was more affected by water availability than nutrient addition and the effect of adding nutrients was not fully realized under low-water availability. Among the different nutrient treatments, +N+P had the greatest effects on *A. nidus* growth and physiology in both watering treatments. Watering treatment changed leaf elemental concentrations but not their ratios (i.e. C:N and N:P). Nutrient addition altered C:N and N:P ratios and increased the concentration of the added elements in leaves, with more pronounced increases in the high-watering treatment. We conclude that the growth of *A. nidus* is more water- than nutrient-limited. When nutrient limitation occurs (i.e. under high-water availability), nutrient co-limitation is stronger than limitation by N or P alone. This result taken together with studies of other epiphytes suggests greater water than nutrient limitation is likely widespread among epiphytic plants. The limited effects of nutrient addition in the low-water treatment suggest that the effect of atmospheric N deposition on epiphyte growth will be limited when water availability is low.

## Teaser

Through a manipulative study in a greenhouse, we illustrated that the growth of an epiphytic fern, *Asplenium nidus* , was more limited by water, and the effect of nutrient limitation is not fully realized when water was limited. We also found that in terms of nutrient limitation, the epiphytic fern was co-limited by nitrogen and phosphorus. If climate change leads to increases in aridity, atmospheric nitrogen deposition will have a limited effect on enhancing the growth of the epiphytic fern.

## Introduction

Epiphytes are a defining feature of tropical and subtropical forests. In some tropical forests, epiphytes contribute up to 30–35 % of plant species richness (Gentry and Dodson 1987; Kϋper *et al.* 2004). Ecologically, epiphytes contribute to canopy water and nutrient retention (Umana and Wanek 2010; Zotz and Winkler 2013; Chen *et al.* 2019; Victoriano-Romero 2020). In addition, some epiphytes such as bromeliads and large epiphytic ferns (e.g. *Asplenium* sp.) provide ample foraging substrates for animals, reproductive habitat, especially for egg-laying animals, as well as refugia against unfavourable weather (Gonçalves-Souza *et al.* 2010; Romero *et al.* 2010; Scheffers *et al.* 2014; Morales-Linares *et al.* 2021). For example, a study in central Panama reported that 3 epiphyte species provided habitat for 89 morphospecies of Anthropoids (Stuntz *et al.* 2002).

The lack of access to soil water and nutrients makes epiphytes more dependent on, and, therefore, potentially more sensitive to changes in, atmospheric inputs of water and nutrients compared to non-epiphyte plants (Benzing 1998; Zotz 2016), although some epiphytes have alternative nutrient sources such as nutrient-providing insects (e.g. ants, Watkin *et al.* 2008; Gegenbauer *et al.* 2023). Global change drivers, including atmospheric nitrogen (N) deposition and reduced water availability because of warming and drought, can have major impacts on plant physiology, growth and survival (Aber *et al.* 1997; Guan *et al.* 2019; Jiao *et al.* 2021). Increased atmospheric N deposition has been shown to decrease the growth and diversity of epiphytic bryophytes (Mitchell *et al.* 2004; Song *et al.* 2012). However, atmospheric N deposition has decreased in many parts of the world including Taiwan over the last several decades (Chang *et al.* 2017; Gilliam *et al.* 2019; Wen *et al.* 2020) and the effect of nutrient reduction on epiphytes that are sensitive to atmospheric nutrient input is largely unknown. While some studies have reported that vascular epiphytes are sensitive to drought including episodic short-term drought in the tropics (Gotsch *et al.* 2018; Williams *et al.* 2020), a recent study found that epiphytes are rather resistant to severe drought events (Einzmann *et al.* 2022). Currently, our understanding of the impact of global change on epiphytes lags far behind that on non-epiphytes, despite their potentially high vulnerability due to the close interaction with atmospheric environment (Lugo and Scatena 1992; Zotz and Bader 2009).

It has been long recognized that the growth of plants can be constrained by nutrient availability. Although the nutrient in the lowest supply is logically the most limiting (Liebig 1840), two main nutrients are understood to limit plant growth globally—N and Phosphorus (P). A recent study reported that at the global scale, N limitation is more prevalent in harsh (cold and/or dry) climates and phosphorus (P) limitation is common in regions with phosphorus-depleted parent materials (Augusto *et al.* 2017). Studies have also shown that warming may decrease P concentration and soil total P pools due to increasing P uptake by plants and output by the transportation of colloidal and particulate P (Peter and Sommer 2015; Tian *et al.* 2023).

Yet, the situation on epiphytes, particularly the co-limitation by N and P is not well understood compared to non-epiphytic terrestrial plants. For example, a fertilization experiment for tank bromeliad *Werauhia sintenisii* (Baker) J. R. Grant in tropical montane cloud forest of Puerto Rico reported a positive growth response to nutrient additions (Lasso and Ackerman 2013). Although the addition of multiple fertilizers simultaneously made it difficult to clearly identify the most limiting nutrient element, based on foliar P concentration and N:P ratio, it is inferred that *W. sintenisii* was P-limited. A study of bromeliad ^15^N isotopic signatures and foliar N and P stoichiometry suggested that P limitation could be widespread in epiphytes (Wanek and Zotz 2011). Additionally, based on results from a fertilization experiment in a montane Hawaiian rainforest, epiphytes might share similar nutrient limitation syndromes to their co-occurring land plant communities (Benner and Vitousek 2007). Compared to the large number of studies on nutrient limitation of non-epiphytic land plants, studies of nutrient limitation of epiphytes are rare and as such generalizations cannot be drawn with confidence.

In addition to nutrients, plant growth is also constrained by water availability. Aridity has increased considerably since 1970 in many parts of the globe including Southeast Asia and is projected to continue to increase (Dai 2011; Vicente-Serrano *et al.* 2020). Water availability oftentimes increases plant access to available nutrients, many of which are acquired in water-soluble forms, so there is an interaction between water and nutrient availability that affects plant physiological functioning (Gregory *et al.* 1997). Numerous studies have evaluated the effects of reduced water availability on plant growth and net primary productivity on a variety of ecosystems including tropical rainforests (Kumagai and Porporato 2012; Corlett 2016; O’Connell *et al.* 2018). Many studies have also examined the effect of nutrient addition via atmospheric deposition on plant growth (Tipping *et al.* 2019; Xing *et al.* 2022). However, few studies have simultaneously manipulated water and nutrient availabilities and examined how they interact to affect plant growth, and based on our knowledge there are no such studies that use epiphytes. To more accurately predict the co-occurring changes in nutrient addition via atmospheric deposition and water availability on plant growth, we need studies that tackle the two important aspects of global change simultaneously.

In this study, we manipulated water and nutrient (N and P) availability to *Asplenium nidus*, an epiphyte common in tropical and subtropical Asia, and examined the effects on plant growth and tissue nutrient concentrations. We tested four hypotheses. First, based on a previous study that reported no response of the growth of *Asplenium* to N and P addition (Huang and Lin 2016), we hypothesized that epiphyte growth is more limited by water availability than nutrients (*H*_1_). Thus, nutrient limitation is weakest when water availability is low (*H*_2_). Second, drought stress typically results in full or partial stomatal closure in most plants. If photosynthesis is reduced because of stomatal closure, CO_2_ cannot be utilized as efficiently, resulting in a decreased ratio of intracellular CO_2_ concentration relative to extracellular CO_2_ concentration (*c*_*i*_/*c*_*a*_). Due to the residence time of CO_2_ in the leaf intercellular air space and the biochemical affinity of Rubisco for the lighter _12_C, a decrease in *c*_*i*_/*c*_*a*_ results in a less negative ^13^C isotope ratio (Farquhar *et al.* 1989). Thus, we hypothesized that ^13^C isotope ratio was greater (less negative) in *A. nidus* with lower water availability (*H*_3_). Third, due to the elemental homeostasis of leaves (i.e. consistent ratios of different nutrient elements), we hypothesized that nutrient addition (N, P) would increase the absolute concentrations of added nutrients (*H*_4_) but would not change their elemental ratios (*H*_5_).

## Materials and Methods

### Plant collection and leaf area determination

*Asplenium nidus* L. (Aspleniaceae) plants were collected between August and September 2021 from the Fushan Experimental Forest (24°45ʹ40″N, 121°34ʹ49″) and the Fushan tribe (24°46ʹ50″N, 121°30ʹ04″) of northern Taiwan. The Fushan Experimental Forest is a subtropical forest with an annual mean temperature of 18.1 °C, mean annual rainfall of 3840 mm, and monthly mean humidity of more than 90 % throughout the year between 1994 and 2013 (Chang *et al.* 2017). The elevation of the Fushan Experimental ranges from 600 m to 1400 m but we collected at elevations lower than 800 m. The Fushan Tribe is approximately 8 km west of the Fushan Experimental Forest and has an annual mean temperature of 19.9 °C and mean annual precipitation of 3910 mm.

*A. nidus*, commonly known as the ‘litter basket epiphyte’ or ‘nest fern’, is among the most abundant vascular epiphytes in low-elevation humid forests in Taiwan and many tropical and subtropical forests in Asia (Freiberg and Turton 2007; Zhang *et al.* 2010). A unique feature of *A. nidus* and several other species of *Aspenium* is the vertical and outward layout of leaves which form a bowl-shaped crown that can intercept canopy litter and other organic matter to form humus-rich substrate (Fayle *et al.* 2008). The size of *A. nidus* varies considerably, with the crown diameter as large as 2 m and leaf length reaching 1.5 m for large plants. We limited our collections to individuals with fully expanded leaves ranging from 20 cm to 75 cm to minimize potential size-dependent variation in plant physiology (Martin *et al.* 2004). All plants were collected from different host trees and any two host trees were at least 3 m apart.

We used a total of 66 mature leaves to establish the relationship of leaf area to leaf length (L) width at 1/4, 2/4, 3/4 of L from the leaf base. Twenty-six leaves were collected from plants in the field that were not used in the experimental treatments and the rest 40 were picked from the experimental plants prior to the treatments. Each leaf was scanned and the area was determined using ImageJ (Schneider *et al.* 2012).

Using backward multiple regression models, we established the following relationship


$$\begin{array}{l} \sqrt{leaf\;area}=\;-0.4185+0.1974L+0.6997\left(\frac{1}{4}\right)L\;+0.4692\left(\frac{3}{4}\right)L\;R^{2}=\;0.954\;\;\;. \end{array}$$

The relationships were used to estimate leaf area of each plant throughout the experiment.

### Experimental design and treatments

The experiment was conducted in a greenhouse located on the Gonguang Campus (24 km north of the Fushan Tribe) of the National Taiwan Normal University in Taipei, Taiwan. Greenhouse temperatures ranged mostly from 25 °C to 31 °C with the lowest being 12.3 °C and highest being 38.8 °C. The relative humidity was relatively stable between 50 % and 70 %.

The experiment was carried out in a factorial design, with two watering treatments—low and high, and four different nutrient addition treatments—control, N addition (+N), P addition (+P) and N and P addition (+N+P). Approximately 60 plants of *A. nidus* collected from two humid forests, the Fushan Experimental Forests and the forest of Wulai of northern Taiwan. The size of *A. nidus* varies considerably, with the crown diameter as large as 2 m and leaf length reaching 1.5 m for large plants. We limited our collections to individuals with fully expanded leaves ranging from 20 cm to 75 cm to minimize potential size-dependent variation in plant physiology (Martin *et al.* 2004). Fifty-six plants were evenly assigned to the eight groups (two watering treatments × four fertilization treatments) with seven plants per group. Each plant was placed into a pot (with an inner diameter of 25 cm and height of 17.5 cm) filled with 150–200 g *Sphagnum palustre* L. peat (a commonly used horticultural growing medium constituent) potting mix. Growing epiphytes in pots is not ideal but it is difficult to maintain their epiphytic growth form in a greenhouse. It is also difficult, if not impossible, to conduct *in situ* experiments that control factors that may confound the treatment effects. Thus, it is not uncommon to conduct controlled experiments of epiphytes using potted plants (Pires *et al.* 2013; Hoeber *et al.* 2020).

The experiment was conducted between October 2021 and September 2022. Prior to the experiment, the plants were watered until saturated once or twice daily. One week before the treatments, leaf number, length and width (as described above) were measured. Plants were assigned into their treatment groups to control for the number of mature leaves, which measured between 60 and 67 in each group. As such there was no significant difference in leaf area (estimated from the relationship between area, length and width, as described above) among the eight groups (One-way ANOVA *F*_(7, 48)_ = 0.2771, *P* = 0.9769). To minimize environmental effects (e.g. light variability within the greenhouse), we randomly rotated plants monthly throughout the experiment period.

The plants in the high-water treatment were watered daily with 150 mL distilled water between spring equinox and fall equinox and 150 mL every other day otherwise. The 150 mL was determined from multiple watering trials, where more than 150 mL would lead to soil saturation (i.e. water dripping from the bottom of pots) and thus the potential leaching of applied fertilizer. Based on our daily observation, the peat potting mix was always moist in the high-water treatment. The plants in the low-water treatment were watered daily with 20 mL distilled water between the spring equinox and fall equinox and 20 mL weekly outside the period. Observationally, there was significant drying of the peat potting mix in the low-water treatment.

For the nutrient addition treatment, fertilizer was applied to the pots (i.e. roots) monthly between October 2021 and September 2022 in the same quantities. Following the methodology of a well-known epiphyte nutrient addition experiment in Hawaiian tropical forests (Benner and Vitousek 2007), we applied the equivalent of 100 kg ha^−1^ yr^−1^ of N and P using Ammonium nitrate (NH_4_NO_3_, Yankuang Lunan Chemical Fertilizer Plant, Shandong, China) for N addition and Sodium Phosphate (NaH_2_PO_4_, Yankuang Lunan Chemical Fertilizer Plant, Shandong, China) for P. In each application, 87.9 mg NH_4_NO_3_ (+N) or 154.7 mg NaH_2_PO_4_ (+P) or a combination of the two (+N+P) dissolved in 15 mL distilled water and applied to their respective nutrient addition treatments, while the control treatment received 15 mL distilled water (without any added nutrients).

We tracked the growth and senescence of individual leaves by giving each leaf a unique number. We counted the number of leaves of each plant and the number of wilting and newly emerging leaves. We measured the length of all mature leaves and randomly selected five leaves to measure leaf length and width at (1/4)L and (3/4)L as described above to calculate leaf area. Therefore, we obtained estimates of monthly leaf areas for each plant.

### Foliar element and carbon isotope analysis

Seven days prior to the treatments, we randomly picked five mature leaves from each of the eight treatment groups for the measurement of foliar nutrient concentrations. At the end of the experiment, all plant leaves were collected. Leaves were cleaned with a paper towel, then measured for fresh mass, followed by oven-drying to constant mass at 45 °C, weighed again for dry mass, and then finely ground using a grinding machine (HsiangTai SM3, Hsiang Tai, Taipei, Taiwan). Leaf tissue of potassium (K), calcium (Ca), magnesium (Mg) and P concentrations were determined via wet digestion of leaf ash in HCl using Inductively Couple Plasma atomic emission Spectrophotometer (ICP-AES JY2000, Edison, USA). Total C and N were determined via dry-combustion using a CHNOS Elemental Analyzer (Elementar vario EL III, Hanau, Germany). To determine if the treatment led to different levels of physiological stress (i.e. changes in leaf gas exchange), we measured leaf carbon isotopic compositions because it can characterize reductions in stomatal conductance and evapotranspiration, and increases in intracellular CO_2_ concentrations over the lifespan of the leaf (Farquhar *et al.* 1989). Approximately, 5 g of each dried and ground sample was weighed into a tin capsule. Carbon isotopic compositions of leaves were determined using an elemental analyzer (Flash 2000, Themo Scientific) coupled with an isotope ratio mass spectrometry (Delta V advantage, Themo Finnigan). The stable isotope ratio is expressed in δ notation as parts per thousand (‰): δ^13^C = [(*R*_sample_^−^*R*_standard_]/*R*_standard_ × 1000], where *R* = ^13^C/^12^C and *R*_standard_ is the ^13^C/^12^C ratio of Vienna Pee Dee belemnite.

### Data analysis

To evaluate the effect of nutrient addition and water treatments on epiphyte leaf growth, we conducted a three-way analysis of variance (ANOVA). Each of the nutrient additions (nitrogen and phosphorus) was treated as a separate factor and all possible interactions were included while time was treated as a covariate. Because the interactions between watering and N addition was significant (see ‘Results’ section), we conducted post-hoc multiple comparisons among different nutrient treatment groups for low and high-water treatments separately using Tukey tests. One-way ANOVA was used to examine differences in leaf tissue elemental concentration among the eight groups before the treatments. Three-way ANOVA was also used to examine if post-experiment leaf tissue elemental concentrations and δ^13^C values were statistically different between water and nutrient addition treatments. If the interaction between water and nutrient addition treatments was significant, Tukey tests were used for comparisons among different nutrient addition treatments for high- and low-water treatments, separately. In the analysis, the leaf areas were expressed as the ratio between total leaf area at the time of measurement relative to the pre-treatment total leaf area. This adjusted plan total leaf area to account for pre-existing differences in plan size prior to the start of the treatments. We used paired-sample T-tests to compare foliar nutrient concentration and δ^13^C value before and after the treatments. All the analyses were conducted using R 4.1.3 (R Core Team 2022).

## Results

During the experiment period, eight *A. nidus* died, six of which were in the low-water treatment (six plants) and two in the high-water treatment. In the high-water treatment, one plant in the +N group and one in the +P group died and in the low-water treatment one in the +N, three in the +P and two in the +N+P group died.

### Leaf area ratio

**Figure 1.**
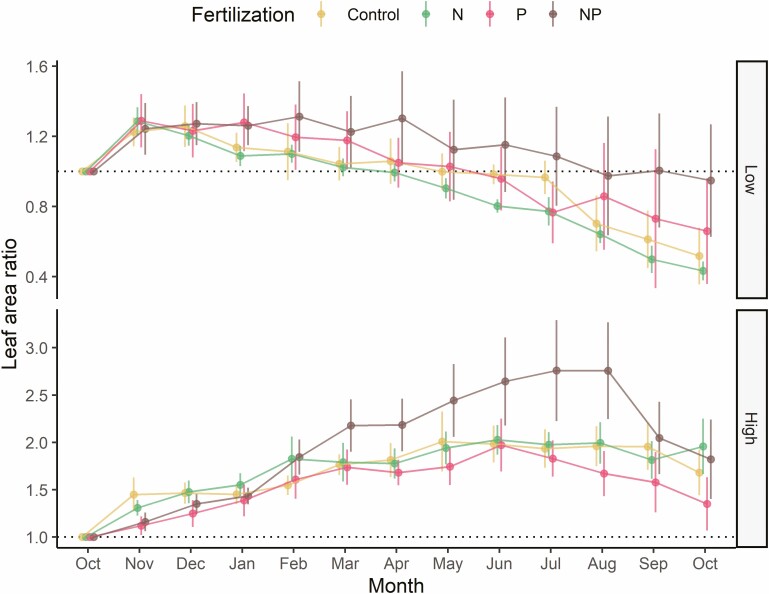
Monthly leaf area ratio (i.e. total leaf area over time relative to the area measured prior to the treatment) for different nutrient addition treatments within the low-water (top) and high-water treatments (bottom). Points above the dotted line (leaf area ratio = 1.0) indicated increases of leaf area compared to prior to the experimental treatment.

Watering, +N, +P, watering × +N and +N × +P had significant effects on leaf area ratio (i.e. total leaf area relative to the measured leaf area prior to treatment) (Table 1). Watering treatment had the biggest effect on leaf production (*F*_(1, 664)_ = 281.02, *P* << 0.001), with mean leaf area ratio being greater in the high-water treatment than the low-water treatment (Fig. 1). Except for the initial increase, leaf area ratio gradually decreased in the low-water treatment over the study period (Fig. 1). However, it gradually increased for the first 7 months then decreased slightly over the last 4 months in the high-water treatment (July–October 2022, Fig. 1). Across all nutrient addition treatments, epiphytes grown under low-water treatment had a mean leaf ratio of 0.96 ± 0.50 (standard error) and epiphytes grown under high-water treatment had a mean leaf ratio of 1.78 ± 0.71.

**Table 1.** Result of three-way ANOVA on the effect of watering, N addition, P addition and their interactions on leaf area ratio (the ratio of the monthly leaf area to the leaf area prior to the treatment) from 56 plants grown over 12 months.

Factor	*F*_(1,664)_	*P*
Watering	281.02	<<0.001
N addition	8.54	0.0035
P addition	9.31	0.0024
Time (month)	4.64	<<0.001
Watering × N addition	5.77	0.017
Watering × P addition	1.533	0.22
N addition × P addition	13.21	<0.001
Watering × N addition × P addition	1.58	0.21

**Figure 2.**
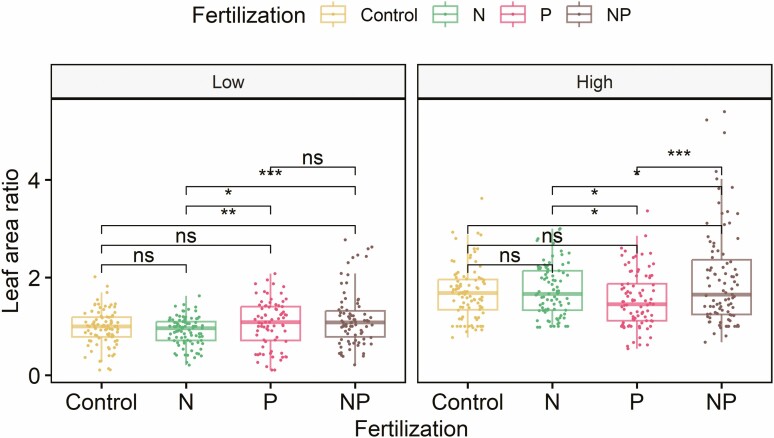
Boxplots of leaf area ratio (i.e. total leaf area over time relative to the area measured prior to the treatment) for different nutrient addition treatments within low-water (left) and high-water treatments (right). *, ** and *** indicate significant difference at *P* < 0.05, 0.01 and 0.001, respectively. ns: not statistically significant.

As described in the ‘Methods’ section, because the interactions between watering and N addition were significant, we compared relative leaf area ratio among different nutrient treatments for high- and low-water treatments separately. In the low-water treatment, leaf area ratio of the control group (0.97 ± 0.04) was only significantly different from (lower than) that of the +N+P group (1.07 ± 0.07) (Fig. 2). Among the three nutrient addition groups within the low-water treatment, leaf area ratio was not significantly different between the +N+P and the +P group (0.92 ± 0.06) but both were significantly greater than that of the +N group (0.89 ± 0.06) (Fig. 2). In the high-water treatment, relative leaf area was also greater in the +N+P group (2.05 ± 0.11) than the control group (1.75 ± 0.06). Among the three nutrient addition groups within the high-water treatment, leaf area ratio was significantly greater in the +N+P group than the +N group (1.77 ± 0.06), which was, in turn, greater than in the +P group (1.56 ± 0.06). Sampling time was also a significant factor affecting leaf area ratio (Table 1) as the differences among treatments were not consistent throughout the study period (Fig. 1).

### Elemental concentration and C:N and N:P ratios

There were no statistical differences in the concentration of any of the analysed leaf tissue elemental concentrations among the eight groups prior to the experiment (all *P* values > 0.05). Over time, the treatments generally did not change the concentration of most elements, including leaf and C:N and N:P ratios (Table 2). The exceptions are: in the low-water treatment, C concentration increased in all but the control group, Ca decreased in the +P group and Mg decreased in the +N and +N+P groups; in the high-water treatment, P concentrations increased in the +P and +N+P groups, Ca increased in the +P group, K decreased in the +N and +P groups and N:P ratio decreased in the +P and +N+P group (Table 2).

**Table 2.** Differences in leaf tissue elemental concentrations and C:N and N:P ratios before treatment application and at the end of the experiment for each of the eight groups. **+** indicates significantly higher and **–** indicates significantly lower at the end of the experiment compared to before the experiment based on paired *T*-test at *P* = 0.05.

Treatment	Low water				High water			
	Control	+ N	+ P	+ NP	Control	+ N	+ P	+ NP
C		+	+	+				
N								
P							+	+
Ca			^−^				+	
Mg		^−^		^−^				
K						^−^	^−^	
C:N								
N:P							^−^	^−^
δ^13^C	+	+	+	+				+

By the end of the experiment, the watering treatment had a significant effect on the concentrations of all analysed elements except for element ratios (N:P), while nutrient addition mainly had significant effects on the leaf tissue elemental concentrations and ratios of those added via fertilization (i.e. N, P and C:N and N:P ratios, Table 3). In addition, the interaction between the watering and nutrient treatments was significant for the concentration of N and P and their ratio (Table 3) and as such the effect of fertilization on leaf tissue elemental concentrations and their ratios was further examined separately for high and low-water treatments.

**Table 3.** Three-way analysis of variance table evaluating the effect of water, N addition, P addition and their interactions on post-treatment leaf tissue elemental concentrations, C:N and N:P ratios and δ^13^C. *n* = 56. *P* values that are statistically significant (<0.05) are in bold.

Element	Watering		N		P		Watering × N		Watering × P		N × P		Watering × N × P	
	*F*	*P*	*F*	*P*	*F*	*P*	*F*	*P*	*F*	*P*	*F*	*P*	*F*	*P*
C	12.25	**0.001**	1.508	0.225	0.069	0.793	0.479	0.492	0.907	0.346	0.181	0.672	0.877	0.353
N	8.745	**0.0048**	34.66	**<0.001**	2.350	0.132	14.08	**<0.001**	2.021	0.162	4.272	**0.044**	0.000	0.996
P	21.83	**<0.001**	0.468	0.497	102.2	**<0.001**	0.857	0.359	41.45	**<0.001**	0.677	0.415	0.939	0.338
Ca	110.2	**<0.001**	0.114	0.737	3.133	0.083	5.917	**0.019**	0.574	0.452	3.141	0.083	0.031	0.861
Mg	15.11	**<0.001**	0.100	0.753	0.148	0.701	1.286	0.262	**0.014**	0.906	0.367	0.548	2.658	0.110
K	11.5	**0.0014**	2.306	0.136	0.264	0.610	0.326	0.571	1.466	0.232	0.215	0.645	0.007	0.932
C:N	4.654	**0.036**	24.15	**<0.001**	0.762	0.381	5.971	**0.018**	0.142	0.708	3.260	0.077	0.260	0.612
N:P	2.578	0.115	26.58	**<0.001**	94.71	**<0.001**	7.455	**0.0088**	28.02	**<0.001**	13.85	**<0.001**	6.133	**0.017**
δ^13^C	5.203	**0.027**	0.142	0.708	0.001	0.973	0.093	0.762	5.717	**0.021**	1.130	0.293	1.725	0.195

**Figure 3.**
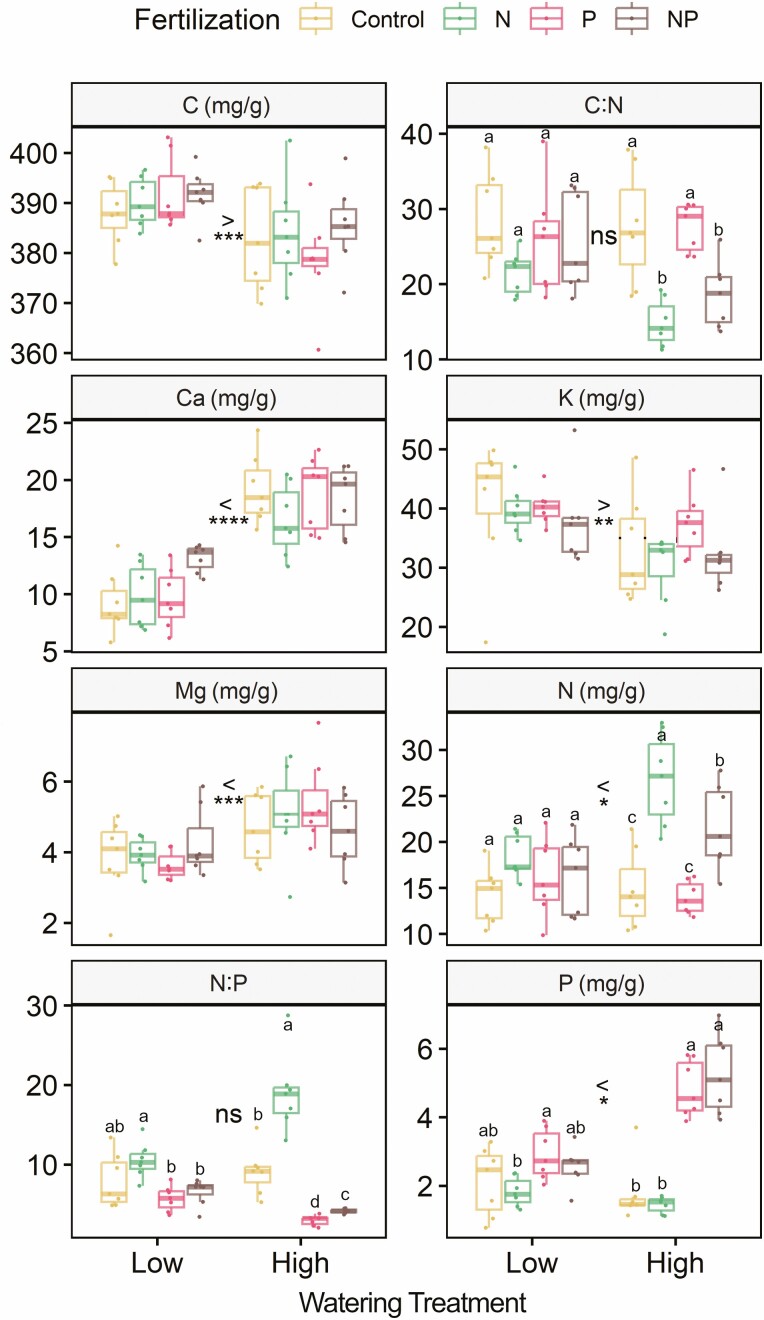
Boxplots of *Asplenium nidus* leaf elemental concentrations of Carbon (C), Nitrogen (N), Phosphorus (P), Calcium (Ca), Magnesium (Mg), Potassium (K) and C:N and N:P ratios by watering and fertilization treatments. Units for each element are given in the panel headings. Statistically significant differences (*P* = 0.05) and their directions between watering treatments are indicated by > or <, where ns. indicates no significant difference. *, ** and *** indicate significant differences at *P* < 0.05, 0.01 and 0.001. Letters denote Tukey test groupings for nutrient addition treatments within watering treatments (i.e. by fertilizer group; note that letters are not comparable across watering treatment groups); where letters are absent, there are no significant differences among nutrient addition treatments.

Comparing high- and low-water treatments, the high-water treatment had significantly higher concentrations of leaf N, P Ca and Mg while the low-water treatment had significantly higher C and K concentrations (Fig. 3).

Among different nutrient addition groups, overall N and P addition increased leaf N and P concentrations but the effect was more pronounced in the high-water treatment than in the low-water treatment (Fig. 3). Leaf tissue N concentrations were significantly higher in the +N and +N+P groups than the other two groups in the high-water treatment while there were no significant differences among nutrient addition groups in the low-water treatment. Similarly, leaf tissue P concentrations were significantly higher in the +P and +N+P groups than in the other two groups in the high-water treatment. In the low-water treatment, however, P concentrations were significantly higher in the +P group than in the +N group. Leaf tissue N:P ratios were significantly different among all groups in the high-water treatment with the +N treatment having the greatest N:P ratios followed by the control, +N+P and +P treatments. In the low-water treatment, the N:P ratios were significantly higher in the +N group than in the +P and +N+P groups. C:N ratios were greater in the control and +P groups than in the +N and +N+P groups in the high-water treatment, but were not significantly different among any nutrient addition groups in the low-water treatment.

### Carbon stable isotope ratio

Compared to pre-treatment values, δ^13^C values increased in the +N+P group of the high-water treatment and for all nutrient addition groups of the low-water treatment (Table 2). By the end of the experiment, δ^13^C value was significantly higher in the low-water (^−^24.73 ± 1.40, mean ± standard deviation) treatment than in the high-water treatment (^−^25.39 ± 0.70) while there were no significant differences among different fertilization treatments (Table 3).

## Discussion

### Water limitation

Using changes in leaf area as a proxy of plant growth, the growth of *A. nidus* was clearly more affected by water availability than by nutrient addition in support of *H*_1_ of greater water than nutrient limitation. Many epiphytic ferns store water in their tissues (e.g. hydrenchyma in *Pyrrosia lanceolata* (L.) Fraw. (Polypodiaceae)) and organs (e.g. pseudobulb in *Nephrolepis cordifolia* (L.) C. Presl (Nephrolepidaceae)), which may help to mitigate water stress (Chiang *et al.* 2013; Rai and Moktan 2023). *A. nidus* has a large substrate that can store large amounts of water. While leaf area increased in the high-water treatment, it decreased in the low-water treatment, suggesting that water storage structures do not necessarily prevent the epiphytic fern from water stress.

The mean leaf area ratio of the +N+P treatment was 2.05 ± 0.11 in the high-water treatment compared to 1.07 ± 0.07 in the low-water treatment indicating a much greater fertilization effect under high-water treatment. The more pronounced response to nutrient addition in the high-water treatment compared to the low-water treatment suggests that alleviation of growth limitation due to nutrient addition is not fully realized under low-water availability, further supporting water limitation. This could probably explain the lack of fertilization effect of in-situ nutrient addition on the growth of two epiphytic ferns (one is a species of *Asplenium*) at the Fushan Experimental Forest of northern Taiwan (Huang and Lin 2016). However, more studies are needed before generalizations can be drawn with confidence.

Although water limitation is more common in dry regions than humid regions, epiphytes may encounter frequent drought even in tropical wet forests (Gotsch *et al.* 2015; Huang and Lin 2016; Martin 2017) because of their lack of direct access to soil water and the fluctuation of microclimate such as solar radiation, vapour pressure deficit and temperature (Fetcher *et al.* 1985; Gotsch *et al.* 2017). In our study, the δ^13^C value of *A. nidus* leaves increased due to reduced water availability which supported *H*_*3*_ and indicated that leaves became more stressed. Water stress is likely a main cause of leaf and whole-plant mortality in *A. nidus.* Our results support this, in that we recorded greater mortality in the low-water treatment (21 %) than in the high-water treatment (7 %).

As a litter-basket epiphyte with a relatively large amount of decomposing organic matter that can store water (estimated as up 6.2 times of its dry weight), *Asplenium* has been suggested to be rather drought-resistant relative to other co-occurring epiphytes such as *Haploperis zosterifolia* (Willd.) E. H. Grane (Jian *et al.* 2013). However, our results show that the growth and survival of *A. nidus* is reduced by prolonged water stress. Hydraulic studies have also indicated that water conservatism is critical for epiphytic ferns to cope with the high-light environment, wind and drying of the canopy habitat (Pittermann *et al.* 2013; Campany *et al.* 2021). We conclude that epiphytes, in general, are sensitive to drought (Gotsch *et al.* 2018; Williams *et al.* 2020). Because global warming is likely to increase drought frequency and severity in many regions including humid tropical regions (Dai 2013), the implication of our results is that *A. nidus* may be increasingly stressed and experience increased mortality and variable population dynamics. Future studies comparing drought responses between epiphytic and non-epiphytic plants of the same species (e.g. *Nephrolepis auriculata* (L.) Trimen) or between epiphytes and closely related non-epiphytes could help to clarify if epiphytes are indeed more vulnerable to drought than non-epiphytes.

### Nutrient limitation

*A. nidus* appears to be co-limited by N and P as leaf area was typically greater in the +N+P group than the others both under the high- and low-water availability throughout the experiment (Figs 1 and 2). Based on a synthesis of 641 studies from diverse ecosystems, Harpole *et al.* (2011) found that more than 50 % of the studies showed some degree of co-limitation by N and P but the synthesis did not include epiphytes. Thus, our study extends nutrient co-limitation to a unique plant group—epiphytes—and thus supports the widespread nature of nutrient co-limitation among diverse plant growth forms.

Leaf area declines in the low-water treatment across all nutrient treatments (Fig. 1) clearly illustrate that in dry conditions, nutrient addition does not improve epiphyte growth supporting the prediction of *H*_*2*_. Our result also points to co-limitation by water and nutrients as has been reported previously for non-epiphytes. Using data of SLA and leaf elemental and isotope analyses, a study along an elevational gradient in Costa indicated that epiphytes are constrained by water and nutrients (N or P) (Cardelús and Mack 2010). A study of an epiphytic bromeliad, *Vriesea sanguinolenta* Cogn. & Marchal in a large forest gap in a lowland forest of Panama reported that the growth of *V. sanguinolenta* was most constrained by water availability but nutrient (N) availability also played a role (Laube and Zotz 2003). In our study, the co-limitation by water and nutrients and the lack of a positive growth response to nutrient addition in the low-water treatment in our study suggest that the potential positive effect of atmospheric N addition on epiphytic plant NPP may not be fully realized in regions that are projected to have reduced water availability. Thus, when modelling the effect of N deposition on NPP (de Vries *et al.* 2014; Tharammal *et al.* 2019), variation in water availability should be taken into consideration. Notably, although the differences in leaf area ratios among different nutrient addition treatments were not entirely consistent during the one-year experiment, the overall patterns were clear for both the high- and low-water treatments (Fig. 1). Given the temporal fluctuation and spatial heterogeneity in water and perhaps nutrient condition, results from a single measurement in time need to be interpreted with caution.

### Response of leaf tissue elemental concentrations and elemental ratios to water and nutrient availabilities

The lack of difference in leaf tissue nutrient concentrations among different groups prior to the experiment (Table 1) suggests that our results are not affected by differences in initial plant tissue nutrient concentrations. Despite the overall lack of treatment effects on nutrient concentrations based on paired comparisons of measurement taken prior to and after the treatments (Table 2), increases in leaf C concentrations for the +N, +P and +N+P groups in the low-water treatment were still observed. The increases in leaf tissue C concentrations in the low-water treatment but not the high-water treatment could be attributed to osmoregulation resulting from increases in concentrations of soluble sugars or other non-structural C compounds that play an important role in plant adaptation to drought stress (Handa *et al.* 1983; Körner 2003).

However, we did find some differences in elemental concentrations among the different nutrient addition treatments by the end of the experiment, mainly for the elements that we added (Fig. 3), supporting *H*_4_. Not surprisingly, N concentrations were higher in plants where N was added and P concentrations were higher in plants where P was added. Importantly, the treatment effect was much greater in the high-water treatment than in the low-water treatment, supporting that nutrient limitation is low when *A. nidus* is water stressed.

The differences in C:N and N:P ratios among different fertilization treatments (Fig. 3) do not support *H*_5_, but rather show that changes in nutrient availability can cause shifts in leaf tissue stoichiometry (Elser *et al.* 2009; Kou *et al.* 2018). By contrast, C:N and N:P ratios were not significantly different between the high- and low-water treatments (Table 3). Many studies and syntheses have reported that drought leads to leaf stoichiometric changes (Sardans *et al.* 2008; Sun *et al.* 2020) but studies examining the stoichiometry of epiphyte tissues in response to changes in water and nutrient availability are rare. Our study indicates that changes in nutrient availability exert a greater influence on tissue stoichiometry of *A. nidus* than water availability does. The paucity of studies on the stoichiometry of epiphytes in response to global change drivers like drought and nutrient addition makes it difficult to generalize about epiphyte stoichiometry and its plasticity to environmental changes.

## Conclusions

Through experimentally manipulating water and N and P availability, we found that water availability greatly affects the growth of *A. nidus*, and increases of *A. nidus* growth because of nutrient addition were dampened when water availability was low. Epiphytic plants are likely more water-limited than they are nutrient-limited; when nutrient-limited, however, it is likely nutrient co-limitation (N and P) that affects plant growth. Therefore, increased climate variability including overall reductions in rainfall and longer droughts due to global change may constrain or compensate for any fertilization effects of atmospheric N deposition on plant growth.

## Data Availability

The data supporting the findings are openly available at KNB repository https://knb.ecoinformatics.org/view/doi:10.5063/F1GH9GDJ.
